# A Psychological Guide to Upper Face Botulinum Toxin Injections: Baseline Emotional Functions of Facial Expressions

**DOI:** 10.1111/jocd.70508

**Published:** 2025-10-21

**Authors:** Alexander G. M. Hopf, Anna Buchheim, Marietta Hopf, Mia Cajkovský, Dirk W. Eilert

**Affiliations:** ^1^ Institute of Psychology University of Innsbruck Innsbruck Austria; ^2^ Villa Dahlem Private Practice Berlin Germany; ^3^ Yuvell Private Practice Vienna Austria

**Keywords:** botulinum toxin, expressive flexibility, facial emotions, facial expressions, facial feedback hypothesis

## Abstract

**Background:**

The upper face is central to human nonverbal communication, with the glabellar complex, forehead, and lateral canthal area signaling core emotions such as anger, sadness, fear, surprise, and joy. Botulinum toxin type A (BoNT‐A) is widely used to modulate muscle activity in these regions, not only reducing dynamic wrinkles but also affecting emotional expression and perception.

**Aims:**

The aim of this narrative review is to synthesize psychological and neurobiological insights into a region‐focused framework, helping clinicians incorporate emotional considerations into BoNT‐A treatments of the upper face.

**Methods:**

A narrative literature review was conducted using PubMed and Scopus, combining the search terms “botulinum toxin”, “facial expression”, “emotion”, “mimicry” and “psychology”. Peer‐reviewed articles addressing facial Action Units (AUs), neural mechanisms, or psychological outcomes were included. Citation tracking and the authors' clinical expertise further informed the synthesis.

**Results:**

The upper face is integral to various facial expressions, with each region contributing distinct signals relevant to intrapersonal experience and interpersonal communication. BoNT‐A can alter these facial expressions, with potential benefits such as mood enhancement, but also possible limitations in conveying authenticity. While neuroimaging and behavioral findings support links between facial feedback and limbic activity, systematic data on region‐specific psychological outcomes remains scarce.

**Conclusions:**

Understanding baseline emotional functions of facial expressions is crucial for clinicians, as these movements shape intrapersonal experience and interpersonal communication. Integrating this knowledge into counseling enables transparent, well‐informed discussions before BoNT‐A application. Future research should systematically evaluate expressive flexibility and patient‐reported satisfaction with emotional communication.

## 1 Introduction

The upper face plays a central role in human emotional expression and interpersonal communication [1]. Studies have shown that the muscles in the upper face are under greater emotional control than those in the lower face [2, 3]. Movements in the glabellar complex, forehead, and lateral canthal region are essential for conveying basic emotions such as anger, sadness, fear, surprise, and joy [4]. These movements are generated by the coordinated action of one or more specific facial muscles, each movement corresponding to defined Action Units (AUs) based on the Facial Action Coding System (FACS) [5]. Extensive research in psychology and behavioral science has explored the functions of these AUs, showing that they are integral to emotion recognition, social signaling, and the regulation of interpersonal interactions [6, 7, 8, 9].

Botulinum toxin type A (BoNT‐A) is widely used in aesthetic medicine to reduce the activity of these muscles, primarily to soften static and dynamic wrinkles. However, reducing muscle activity, particularly in the upper face, also modulates facial expressions [10]. These expressions are critical channels for communicating emotions, both consciously and subconsciously [1]. Altering them has the potential to change how emotions are signaled to others and how the treated individual is perceived [11]. In addition to these interpersonal effects, such changes may influence the individual's own emotional experience [12, 13, 14], as proposed by the facial feedback hypothesis [15, 16]. For this reason, both patients and clinicians should be aware of the broader functional consequences of modifying facial expressions.

Most existing literature on BoNT‐A in the upper face focuses on aesthetic outcomes or global psychosocial measures, such as improvements in self‐esteem or reduction in depressive symptoms [17, 18]. While important, less research has focused on a related key question: what does each upper facial muscle communicate under normal, untreated conditions, and how might this signaling change when its activity is reduced? Addressing this question is essential for a more nuanced, psychologically informed approach to treatment, essentially considering not only the visible aesthetic results but also the subtler implications for social interactions, emotional authenticity, and emotional experience [19].

The objective of this narrative review is to provide clinicians with a region‐specific, psychological guide to upper‐face BoNT‐A treatments. For each major region, we describe the relevant AUs and their related muscles (see Table 1), outline their normal role in emotional expression, and discuss potential considerations when reducing their activity. By integrating insights from behavioral research and psychology, this framework aims to support more informed clinical decision‐making and enhance the quality of patient consultations.

**TABLE 1:** Overview of selected facial Action Units (AUs) with their primary muscular basis and the corresponding facial expressions involved.

Action unit (AU)	Muscles involved	Movement
AU1	M. frontalis (medial part)	Inner brow raised
AU2	M. frontalis (lateral part)	Outer brow raised
AU4	M. corrugator supercilii, M. depressor supercilii, M. procerus	Brows lowered and drawn together
AU6	M. orbicularis oculi (orbital)	Eye corners tighten; lower lid elevates; eye aperture narrows
AU12	M. zygomaticus major	Lip corners pulled up

## 2 Materials and Methods

A narrative literature review was conducted to synthesize current knowledge on baseline emotional functions and discuss potential psychological and neurobiological implications of botulinum toxin type A (BoNT‐A) in the upper face, with particular emphasis on its effects on facial expressions.

**FIGURE 1:**
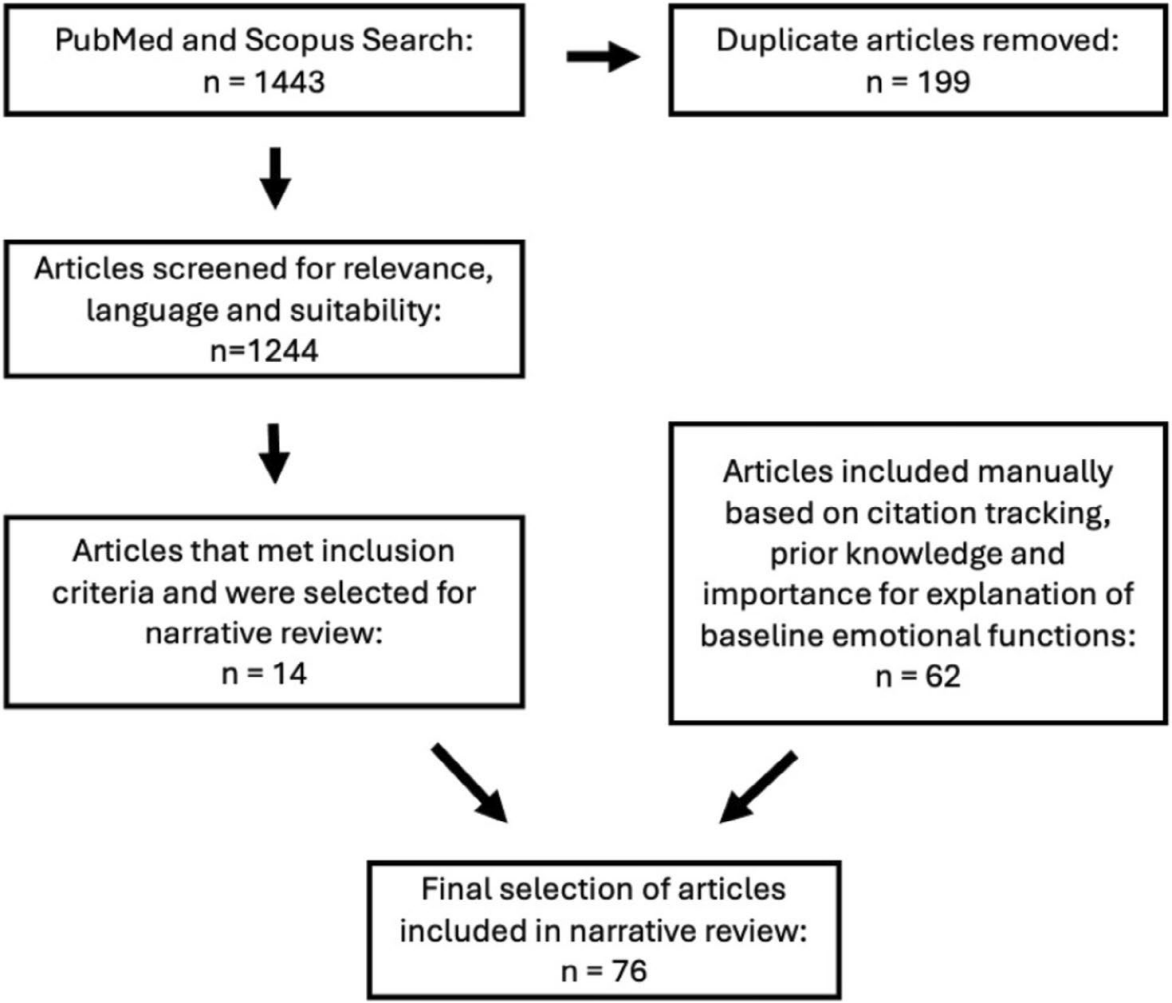
Flowchart depicting PubMed/Scopus search strategy and inclusion criteria. Two evidence streams informed this narrative review: (i) the BoNT‐A evidence stream identified through database screening, and (ii) region‐specific baseline emotional function literature incorporated through citation tracking and prior knowledge.

A PubMed and Scopus search was performed (inception to September 2025) using combinations of the terms “botulinum toxin” AND “facial expression” OR “emotion” OR “mimicry” OR “psychology”. Inclusion criteria were peer‐reviewed, English‐language human studies investigating upper‐face BoNT‐A (glabellar, forehead, lateral canthal regions) with psychological, interpersonal, or neuropsychological insights providing relevant to baseline emotional functions. Exclusion criteria were non‐English or non‐peer‐reviewed publications, animal or in vitro studies, and BoNT‐A in non‐facial or lower‐face regions. Additional references were identified through citation tracking and the authors' clinical expertise in aesthetic medicine and emotion research. A simplified flow diagram (Figure 1) summarizes the identification, screening, and inclusion process for the evidence stream.

Findings were synthesized narratively to highlight baseline emotional functions of upper‐facial expressions, exploring the hypothetical impact of BoNT‐A on facial expressions and providing an outlook on emerging evidence linking aesthetic interventions to psychological outcomes. The description of baseline emotional functions of specific upper‐face Action Units was based on a previous framework [9], which offers a comprehensive reference for linking AUs to emotional signaling.

For manuscript preparation, ChatGPT‐5 was used exclusively for grammar and language editing. All scientific content and interpretations were developed by the authors.

## 3 Results and Discussion

### 3.1 The Glabellar Complex

#### 3.1.1 Baseline Emotional Functions of Facial Expressions

**FIGURE 2:**
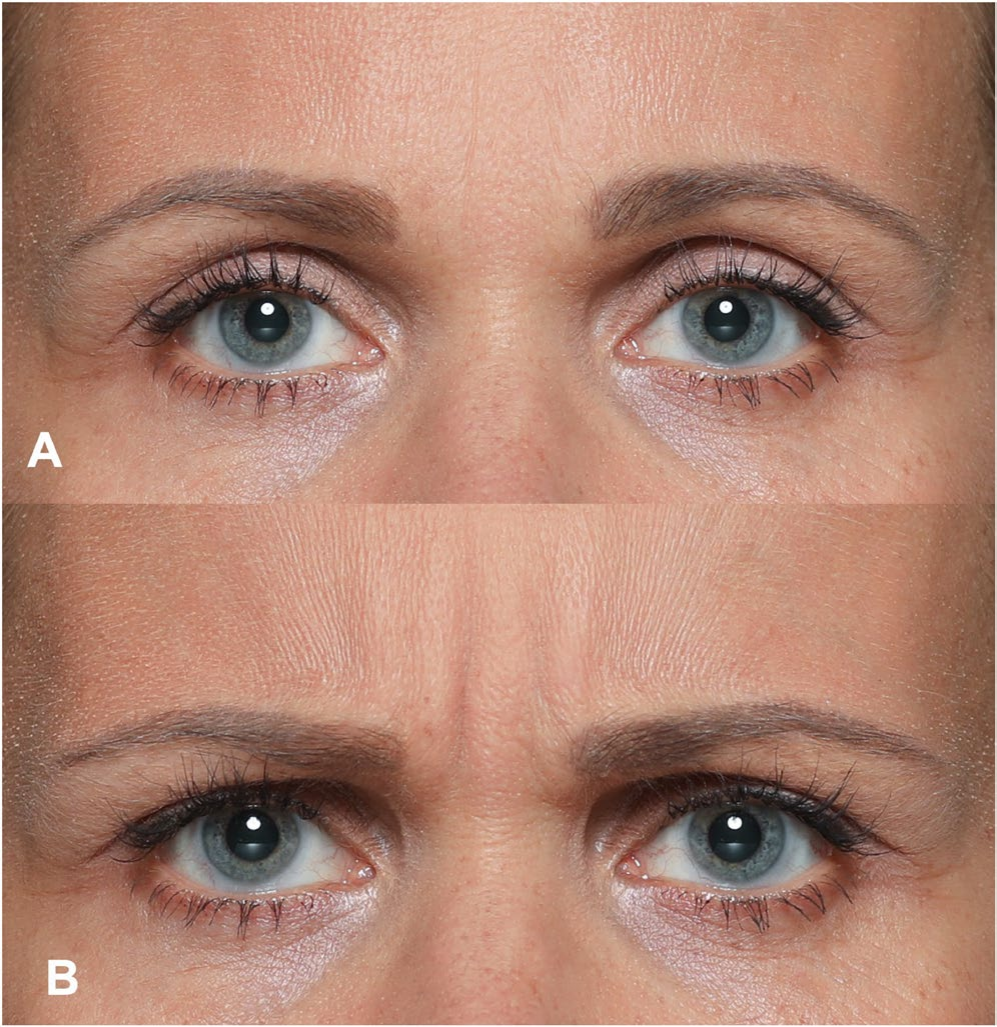
Illustrative photographic depiction of the glabellar complex at rest (A) compared to activation (B) leading to a medial and downward movement of the eyebrows, intensifying glabellar lines.

The glabellar complex consists primarily of the corrugator supercilii, procerus, and depressor supercilii muscles. Together, these muscles are responsible for drawing the eyebrows medially and downward, producing vertical lines between the brows and horizontal creases over the nasal bridge [20]. In FACS, this movement corresponds to AU4 (Brow Lowerer), a core movement of several facial emotions, most notably anger (see Figure 2). Under untreated conditions, contraction of the muscles of the glabellar complex plays a key role in conveying a range of affective, cognitive, and motivational states (see Table A1):

- Anger: Both “hot” (overt, high‐intensity) and “cold” (restrained, low‐intensity) anger expressions frequently involve AU4, often accompanied by raising of the upper eyelids [4].
- Irritation and confusion: Subtle frowning may convey mild displeasure or cognitive uncertainty [4, 21].
- Concentration: Prolonged frowning can serve as a visual cue for mental focus [22].
- Skepticism: Often paired with a slight backward head tilt, frowning may communicate doubt [9].
- Pain: AU4 is among the core movements consistently associated with pain expression [23, 24].
- Effort: The movement is common during physical strain, both in endurance and strength tasks [25, 26].
- Triumph: AU4 is part of the facial core movements to express triumph in a context of competition [27].
- Conversational Emphasis: Frowning is often used to emphasize something when speaking or to emphasize the end of a sentence [28].

#### 3.1.2 Interpersonal Effects

For observers, the perception of intense glabellar lines—caused by AU4—can have a strong social impact. It may be perceived as dominant, threatening, or dismissive [21, 29]. Experimental work has shown that when we make eye contact while frowning at someone, stress levels in the person perceiving this expression rise promptly [30]. In task‐oriented settings, it can convey determination and assertiveness [31]. These interpersonal perceived attributes of the AU4 are supported by the finding that individuals high in agreeableness tend to frown less in social interactions [32].

AU4 functions as a social counterpoint to the smile (AU12), signaling disagreement or disapproval much as a smile signals warmth and acceptance [33].

#### 3.1.3 Neuropsychological Insights

Research has demonstrated that even minimal activation of the glabellar muscles can alter neural processing of emotional stimuli. In a study by Hennenlotter, Dresel, Castrop, Ceballos‐Baumann, Wohlschlager, and Haslinger [34], simply pulling the eyebrows together increased amygdala activation, a brain region central to emotional evaluation and the generation of fight‐or‐flight responses [35, 36].

In addition, several studies have shown that targeted BoNT‐A treatment of the glabellar complex can lead to significant improvements in depressive symptoms. The reported antidepressant effects of BoNT‐A have been observed across different patient populations, including cases resistant to conventional treatments, making them a promising area of psychodermatological and neuropsychiatric research [17]. Nevertheless, a major limitation of the current evidence is the heterogeneity of existing trials with respect to sample sizes, placebo control, and individual injection protocols.

Possible reasons for the observed effects include facial feedback mechanisms and downstream effects on limbic system activity [14, 37, 38]. Evidence from different experimental studies supports the presence of facial feedback effects. For example, Larsen et al. demonstrated that unobtrusive activation of the corrugator supercilii (induced by a “golf tee” task) increased reported sadness when participants viewed aversive photographs. These findings indicate that facial muscle contractions can amplify ongoing unpleasant affect even outside of conscious awareness, supporting the notion that facial feedback contributes causally to emotional experience [39]. Furthermore, recent work by Okazaki, Suzuki, and Duncan [40] demonstrated that activation of the zygomaticus major muscle during smiling can inhibit corrugator supercilii activity both through facial feedback–related positive affect and via short‐term inhibitory mechanisms such as feedforward control, reducing frown expression even before the smile is fully formed.

#### 3.1.4 Key Considerations for Counseling

Targeting the glabellar complex with BoNT‐A reduces AU4 activity, softening frown lines and often enhancing social approachability. Clinical trials also suggest potential mood benefits through facial feedback mechanisms. At the same time, limiting brow mobility can reduce signals of focus, skepticism, or assertiveness.

### 3.2 The Forehead Region

#### 3.2.1 Baseline Emotional Functions of Facial Expressions

**FIGURE 3:**
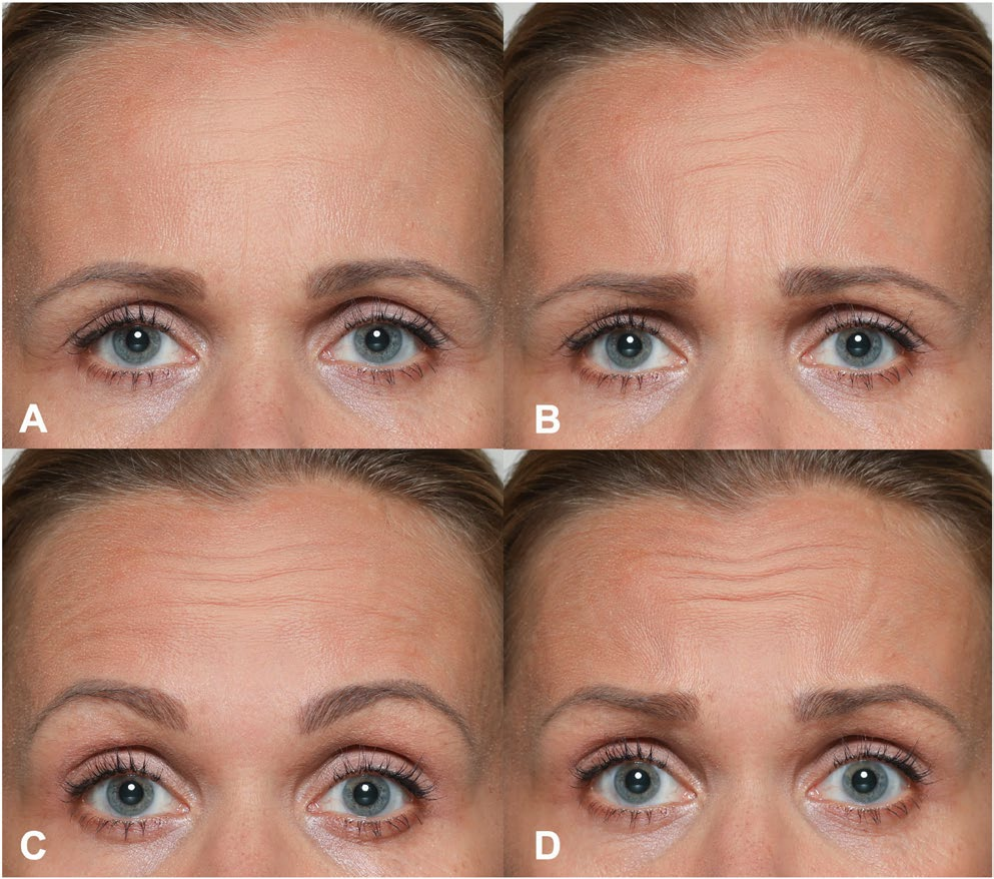
Illustrative photographic depiction of the differentiation of three different upper face expressions involving the frontalis muscle. (A) Neutral; (B) inner brow raiser (AU1); (C) full brow raise (AU1+2); (D) brow raise with brows drawn together (AU1+2+4).

The forehead region is primarily moved by the frontalis muscle, the only elevator of the eyebrows [41]. Unlike the glabellar complex, which is dominated by a single key Action Unit (AU4), the forehead can produce several distinct movements—AU1 (Inner Brow Raiser), AU2 (Outer Brow Raiser), AU1+2 (full brow raise), and AU1+2+4 (full brow raise with brows drawn together) [5, 42]. The Action Units 1, 2, and 4 have fundamentally different baseline emotional functions, depending on whether they occur alone or in combination, making it essential to examine the different facial expressions individually (See Table A2). Figure 3 displays the difference in the expressions of AU1, AU1+2, and AU1+2+4.

##### 3.2.1.1 AU1—Inner Brow Raiser

- Sadness: Can indicate sadness [43].
- Shame: AU1 in combination with other nonverbal movements like gaze aversion [44, 45].
- Guilt: Can convey guilt [46].
- Compassion: AU1 in combination with other nonverbal movements like a slight head movement forward to signal social approach [47].
- Being moved: When combined with a Duchenne smile (AU6+12), it can signal a blend of joy and sadness, often described as being moved [9].
- Awe: When co‐activated with upper eyelid raising (AU5) and jaw drop (AU26), contributes to the awe display [48].

##### 3.2.1.2 AU2—Outer Brow Raiser (Often Unilateral)

- Confusion: Can signal confusion [22].
- Skepticism: Evaluative doubt [49].
- Contempt: Often accompanied by a slight head tilt backward or eyeroll [50, 51, 52].
- Playfulness: Rapid repetitions may indicate flirting or teasing [9].

##### 3.2.1.3 AU1+2—Full Brow Raise

- Surprise: Core movement of surprise [49].
- Interest: Heightened attentiveness or curiosity [53].
- Uncertainty: Sometimes signals uncertainty or doubt, especially when the eyebrow raise is strong [54].
- Conversational Emphasis: Can mark key points or social acknowledgment while speaking [28].
- Greeting: Short eyebrow flashes signal across cultures an invitation for social communication [55].

##### 3.2.1.4 AU1+2+4—Brow Raise With Brows Drawn Together

- Fear: Can indicate fear [43, 56].
- Concern: AU1+2+4 without other movements is a potential signal for worry or apprehension [43, 56].
- Facial distinction from Sadness: While AU1 in sadness typically produces a pronounced upward curve of the inner brow with a visible medial arch, AU1+2+4 in fear or concern, the eyebrows often appear straighter, maybe slightly curved.

#### 3.2.2 Interpersonal Effects

Inner brow raises (AU1) can evoke warmth, compassion, and approachability, but may also signal vulnerability, lowering perceived dominance [9, 57].

Outer brow raises (AU2) can be perceived interpersonally as ironic detachment, doubt, or superiority, while rapid, playful AU2 movements may convey flirtation or teasing [9, 58].

Full brow raises (AU1+2) generally increase perceived openness and attentiveness, enhancing social engagement [59].

The AU1+2+4 pattern conveys urgency or concern, prompting heightened alertness in observers, and is particularly common in safety‐critical contexts.

#### 3.2.3 Neuropsychological Insights

AU1 activation has been associated with vagus nerve engagement and parasympathetic activation, linking it to caregiving and affiliative behaviors [60]. Similarly, AU1+2 has been reported to broaden the subjective visual field and may facilitate faster visual information intake [55], as has the combined activation of AU1+2+4 [61]. These effects, however, were observed in single experimental studies in untreated individuals, and no clinical data currently demonstrate that forehead BoNT‐A produces such neural or perceptual outcomes.

#### 3.2.4 Key Considerations for Counseling

BoNT‐A in the forehead reduces frontalis activity, making AU1, AU2, AU1+2, and AU1+2+4 expressions less pronounced. Potential advantages include reducing habitual tension displays (e.g., persistent “concern” expressions) and improving congruence between felt and displayed affect in patients who report an unintended worried appearance [62]. Potential unwanted psychological implications are a reduced ability for conveying compassion (AU1), evaluative doubt or playful irony (AU2), straightforward openness or acknowledgment (AU1+2), and urgency or concern (AU1+2+4). It is important to note that these potential implications are hypothetical and that studies investigating the actual interpersonal effects of modulating frontalis activity through BoNT‐A are required.

In addition, potential adverse effects such as eyebrow ptosis or compensatory muscle activation can further alter facial expressivity and thereby influence how patients are perceived in social interactions. Such side effects may unintentionally modify emotional signaling, making it important to address them in patient counseling and to investigate their psychological consequences in future research.

### 3.3 Lateral Canthal Lines

#### 3.3.1 Baseline Emotional Functions

**FIGURE 4:**
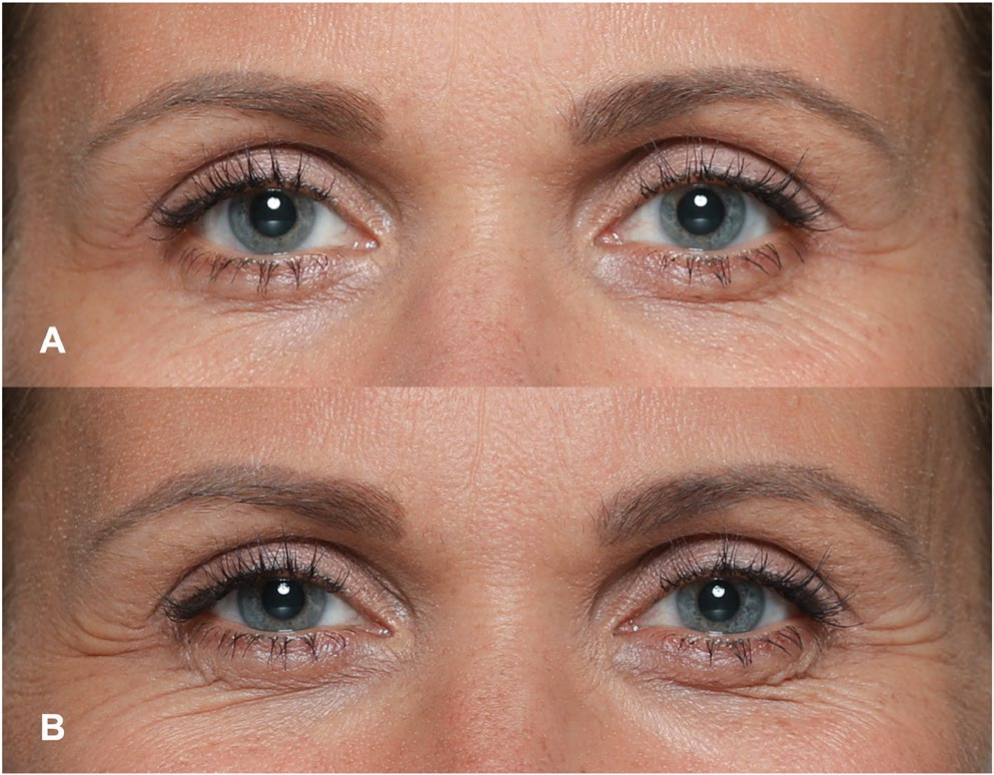
Illustrative photographic depiction of the lateral canthal region when performing a social smile (A) vs. when performing an authentic (Duchenne) smile (B).

The lateral canthal region, often referred to as “crow's feet,” is primarily shaped by the outer portion of the orbicularis oculi muscle. Lateral canthal lines form through concentric contraction around the eyes, narrowing the eye aperture [63]. Accurate identification of this movement (classified as AU6, so‐called Cheek Raiser and Lid Compressor) relies on more than the presence of fine radial lines at the outer corners. It also typically involves the skin at the temples being drawn medially, the upper eyelid fold lowering, a slight lateral brow drop, the infraorbital triangle lifting, and the deepening of the lower eyelid furrow [5]. These changes together reduce the visible aperture of the eye (see Figure 4). While crow's feet can occur in strong Non‐Duchenne smiles, these periocular changes provide a more reliable indicator of AU6 activation [9]. The lateral canthal region is central to several important emotional and social signals (See Table A3):

- Enjoyment and the Duchenne‐Smile: When AU6 appears together with AU12 (M. zygomatic major), it forms the Duchenne smile: A reliable marker of genuine enjoyment [64, 65].
- Love and pride: Combined with a head tilt to the side (love) or backward (pride), the conveyed meaning of AU6+12 changes [66].
- Being moved: When combined with AU1 (Inner Brow Raiser), AU6+12 can signal being moved, a combination of joy and sadness [9].
- Pain expressions: AU6 also appears in the core movement set for pain [24], showing that its presence is not limited to pleasant emotions.

#### 3.3.2 Interpersonal Effects

Activation of AU6 increases perceptions of likability and approachability, and fosters trust and cooperation in social and professional settings [67]. Smiles incorporating AU6 (Duchenne smiles) are more likely to elicit positive reciprocity and emotional engagement from others. Smiling with contraction of the lateral orbicularis oculi muscle increases perceived attractiveness [68]. By contrast, “social smiles” (smiling without AU6) can appear polite but less emotionally genuine, often perceived as more formal or strategic [69]. The absence of AU6 in pleasant expressions may therefore reduce perceived warmth and limit the depth of interpersonal connection.

Pleasant expressions, which involve AU6 (e.g., genuine happiness), also carry strong contagion effects: viewing Duchenne smiles can increase the observer's positive mood [70]. Observers attribute positive traits, such as naturalness, sociability, and friendliness, to people whose smiles include AU6 [67], and are more likely to cooperate with them [71].

#### 3.3.3 Neuropsychological Insights

Neuroimaging studies have shown that producing and perceiving smiling expressions activates reward‐related regions such as the ventral striatum and orbitofrontal cortex [72]. The spontaneous and limbically driven nature of AU6 makes it more resistant to voluntary control, reinforcing its role as a reliable cross‐cultural signal of genuine emotion [65]. Evidence from the Many Smiles Collaboration (including 3878 participants across 19 countries) supports the presence of small facial feedback effects, showing that both facial mimicry and voluntary facial action tasks can increase reported happiness. These findings suggest that facial feedback can amplify ongoing pleasant affect or even initiate feelings of happiness in otherwise neutral contexts, while its magnitude varies depending on the specific task and situational factors [16].

A pilot study by Lewis [14] suggested that combining glabellar and lateral canthal BoNT‐A treatment might attenuate the antidepressant benefit otherwise observed with isolated glabellar injection. These findings must be replicated by large‐scale randomized‐controlled trials to establish robust clinical recommendations.

#### 3.3.4 Key Considerations for Counseling

BoNT‐A treatment in the lateral canthal region effectively smooths crow's feet and can harmonize the periorbital appearance with rejuvenation in other facial areas. However, over‐reduction of AU6 activity may reduce the capacity for Duchenne smiles, subtly affecting warmth and authenticity in social communication. Quantitative evidence supports this concern: Etcoff et al. showed that after BoNT‐A injection into the lateral orbicularis oculi, the proportion of smiles displaying AU6 decreased from 97% before treatment to 19% afterwards. Independent raters further judged these post‐treatment smiles as less “felt”, “spontaneous” and “happy” indicating that the expressive quality of smiles can be perceptibly altered [69].

## 4 Conclusion

Upper‐face expressions targeted with botulinum toxin, including the region of the glabellar complex, forehead, and lateral canthal area, have baseline emotional functions that are integral to social interaction and interpersonal communication [73]. Each region conveys specific non verbal signals that shape both how emotions are perceived by others and how they are internally experienced by the patient [8]. A thorough understanding of these functions prior to intervention is essential to anticipate potential psychological effects and to provide patients with transparent, well‐informed counseling.

Despite an expanding body of research on the interactions of BoNT‐A and facial expressions, this review contains various limitations. The actual impact of upper‐face treatment on patients' expressive flexibility and social communication has not been widely or systematically studied. Most evidence, including the considerations in this review, is extrapolated from studies on baseline emotional functions of facial expressions [9], while controlled clinical data on region‐specific effects after botulinum toxin treatments are scarce. In this context, the counseling perspectives discussed in the review should be understood as hypothetical, reflecting inferences from baseline emotional functions of facial expressions rather than outcomes directly demonstrated in BoNT‐A studies. Furthermore, it is not yet clear whether different botulinum toxin formulations (varying in several key aspects such as diffusion and duration of effect) or alternative injection protocols, such as modified dosing or point distribution, yield distinct psychological outcomes. Addressing these gaps requires systematic research that links BoNT‐A treatment variables not only to aesthetic endpoints but also to the subtler dimensions of emotional expressivity. Future studies may benefit from integrating targeted measures such as validated scales of expressive flexibility and patient‐reported satisfaction with emotional communication, rather than relying solely on global psychosocial outcomes like depression scores.

In addition, future research should investigate how BoNT‐A interventions may shape intrapersonal experience and interpersonal communication in dyadic contexts, particularly with regard to attachment‐related processes. Prior studies have shown that individual attachment representations influence both emotion regulation and expression, as well as intra‐ and interpersonal emotion perception [74, 75]. All of these are core functions of emotion processing (C‐FEP) [76] that are also potentially influenced by BoNT‐A interventions.

Clinically, these uncertainties emphasize the need for careful, individualized treatment planning, with dosing strategies tailored to patient goals and communicative needs [19]. Particularly for first‐time patients, a stepwise, conservative approach, for example, starting with lower doses and reassessment at follow‐up, may help safeguard expressive flexibility while progressively addressing aesthetic concerns. At this moment, these recommendations should be regarded as expert‐opinion guidance, as comparative clinical data are currently lacking. Future studies will be important to determine whether different dosing or injection strategies indeed influence the balance between aesthetic benefit and the preservation of facial communication. Overall, this research will contribute to aligning aesthetic outcomes with long‐term psychological well‐being.

## Author Contributions

A.G.M.H., A.B., M.H., M.C., and D.W.E. were all involved in the conceptualization and planning of the manuscript. A.G.M.H. drafted the initial manuscript. A.B., M.H., M.C., and D.W.E. revised and contributed to the individual sections of the manuscript. All authors approved the final version of the manuscript.

## Disclosure

A.G.M.H. is Director of International Professional Education at Evolus Inc., Newport Beach, California, USA.

## Ethics Statement

The authors confirm that the ethical policies of the journal, as noted on the journal's author guidelines page, have been adhered to. No ethical approval was required as this is a review article with no original research data.

## Consent

Written informed consent was provided by all identifiable people in the photographs.

## Conflicts of Interest

A.G.M.H. is an employee of Evolus Inc., Newport Beach, California, USA. M.H. is a speaker for Croma Pharma, Leobendorf, Austria. The other authors declare no conflicts of interest.

## Data Availability

No datasets were generated or analyzed in this study.

## Appendix

**TABLE A1:** Key considerations for the treatment of the glabellar region, including the baseline emotional functions and potential changes with reduced activity of AU4.

Action units	Baseline emotional functions of facial expressions	Potential changes with reduced activity
AU4 (Brow lowerer)	Anger (hot/cold), irritation, confusion, concentration, skepticism, pain expression, effort signaling, assertiveness/order motives	Softer, more relaxed appearance; reduced perception of dominance or hostility; possible dampening of pain and effort cues; potential mood improvement and antidepressant effects; slight reduction in ability to signal doubt or intense focus

**TABLE A2:** Key considerations for the treatment of the forehead, including the baseline emotional functions and potential changes with reduced activity of the different AUs, expressed in the forehead region.

Action units	Baseline emotional functions of facial expressions	Potential changes with reduced activity
AU1 (Inner brow raiser)	Sadness, shame, guilt, compassion, being moved, awe	Reduced capacity to convey empathy, sadness, compassion, awe
AU2 (Outer brow raiser)	Confusion, skepticism, contempt, playfulness, dominance	Diminished ability to express evaluative doubt, playful teasing, subtle contempt cues
AU1+2 (full brow raise)	Surprise, interest, uncertainty, conversational emphasis, greeting	Slight reduction in perceived openness, attentiveness, social acknowledgment
AU1+2+4 (full brow raise with brows drawn together)	Fear, concern, intense focus, vigilance	Possible dampening of urgency/concern signals, reduced threat‐related cueing

**TABLE A3:** Key considerations for the treatment of the lateral canthal region, including the baseline emotional functions and potential changes with reduced activity of AU6.

Action units	Baseline emotional functions of facial expressions	Potential changes with reduced activity
AU6+AU12 (Duchenne smile)	Genuine enjoyment, warmth, affiliative intent	Decreases perceived likability, reduces trust and cooperation
Duchenne smile + head tilt (side)	Love, affection	Reduces relational closeness
Duchenne smile + head tilt (back)	Pride	Reduces perceived authenticity of pride expressions
Duchenne smile combined with AU1	Being moved	Reduces social warmth and empathy
AU6 in pain expressions	Physical or emotional pain	Decreases signals of need for care or assistance
